# Three TF Co-expression Modules Regulate Pressure-Overload Cardiac Hypertrophy in Male Mice

**DOI:** 10.1038/s41598-017-07981-4

**Published:** 2017-08-08

**Authors:** Yao-Ming Chang, Li Ling, Ya-Ting Chang, Yu-Wang Chang, Wen-Hsiung Li, Arthur Chun-Chieh Shih, Chien-Chang Chen

**Affiliations:** 1 Biodiversity Research Center, Academia Sinica, Taipei, Taiwan; 2 Institute of Biomedical Sciences, Academia Sinica, Taipei, Taiwan; 3 Department of Ecology and Evolution, University of Chicago, Chicago, IL 60637 USA; 4 Institute of Information Science, Academia Sinica, Taipei, Taiwan

## Abstract

Pathological cardiac hypertrophy, a dynamic remodeling process, is a major risk factor for heart failure. Although a number of key regulators and related genes have been identified, how the transcription factors (TFs) dynamically regulate the associated genes and control the morphological and electrophysiological changes during the hypertrophic process are still largely unknown. In this study, we obtained the time-course transcriptomes at five time points in four weeks from male murine hearts subjected to transverse aorta banding surgery. From a series of computational analyses, we identified three major co-expression modules of TF genes that may regulate the gene expression changes during the development of cardiac hypertrophy in mice. After pressure overload, the TF genes in Module 1 were up-regulated before the occurrence of significant morphological changes and one week later were down-regulated gradually, while those in Modules 2 and 3 took over the regulation as the heart size increased. Our analyses revealed that the TF genes up-regulated at the early stages likely initiated the cascading regulation and most of the well-known cardiac miRNAs were up-regulated at later stages for suppression. In addition, the constructed time-dependent regulatory network reveals some TFs including *Egr2* as new candidate key regulators of cardiovascular-associated (CV) genes.

## Introduction

Many heart related diseases, such as ischemic heart disease, hypertension, heart failure, and valvular heart disease, are accompanied by cardiac hypertrophy^1^. This type of cardiac hypertrophy, also called pathological hypertrophy, is associated with altered cardiac gene expression, fibrosis, and cardiac dysfunction and can progress to heart failure eventually. In contrast, the other type of cardiac hypertrophy is physiological and occurs in response to chronic exercise training that is reversible and characterized by normal cardiac morphology without fibrosis or apoptosis^2^. The gene regulation network involved in the hypertrophic process is still largely unknown. Studies using animal models have identified a few genes and regulators and verified some of their specific roles in cardiac hypertrophy^3–13^. The development of cardiac hypertrophy is a dynamic process, and a few studies have taken this fact into consideration^14–16^. For example, Friddle *et al*.^14^ conducted a time course experiment, taking a total RNA sample from heart tissue of the drug-induced cardiac hypertrophy each day in two weeks, and identified dozens of genes associated with cardiac hypertrophy. Van den Bosch *et al*.^16^ conducted another time course experiment from aortic banding-induced cardiac hypertrophy at five time points in 8 weeks and found many differentially expressed genes associated with metabolic activity for compensated hypertrophy. In addition, Sheehy *et al*.^15^ compared the temporal change of transcriptomes measured in hearts subjected to either exercise-induced or aortic banding–induced hypertrophy at different time points. As most studies took cardiac samples only at few time points, a comprehensive study of how the transcription factors (TFs) dynamically regulate the associated genes during the hypertrophic process is still lacking.

Noting that heart undergoes morphological and electrophysiological changes to overcome a pressure overload, Meerson and colleagues divided cardiac remodeling into three stages: developing hypertrophy, compensatory hypertrophy, and overt heart failure^1, 17^. However, how these morphological and electrophysiological changes are regulated is still largely unknown. In this study, we raised three questions: (1) Which genes are involved in the hypertrophic process, from developing hypertrophy to compensatory hypertrophy and to overt heart failure stages? (2) Which TFs or miRNAs are responsible for inducing these changes and are they co-regulated and modularized? (3) What roles do these TFs or miRNAs play in stress-induced cardiac hypertrophy? To answer these questions, we first conducted a time course experiments to collect mRNA and miRNA expression data at five different time points from thoracic aortic banding (TAB)- and sham-operated (control) mouse hearts. Then, we analyzed the time-course transcriptome data and integrated them with the regulatory information from public bioinformatics databases. Based on the results, we attempted to answer the above questions one by one and identified the candidate TFs and miRNAs at different stages of cardiac hypertrophy.

## Results

### Animal model of cardiac hypertrophy, experimental design, and transcriptome data processing

Adult male C57BL/6 J mice were anaesthetized and subjected to pressure overload by transverse aortic banding (TAB) according to the method described previously^18, 19^. The RNAs of cardiac tissues were taken from the left ventricles of mouse hearts subjected to TAB or sham operation at days 3, 5, 7, 14, and 28 after surgery (Suppl. Fig. S1). The expression changes of mRNAs and miRNAs were measured by cDNA and miRNA microarrays, respectively. After the data processing and within and between sample normalizations (see Methods), the total numbers of genes and miRNAs for this study are 16,152 and 928, respectively. The expression fold-changes of each gene and miRNA at each time point were separately calculated by the log2 ratio of the normalized intensity values with and without the pressure overload treatment (Suppl. File S1).

### Day 5 is the major transitional stage of transcriptome expression after pressure overload in mouse hearts

**Figure 1:**
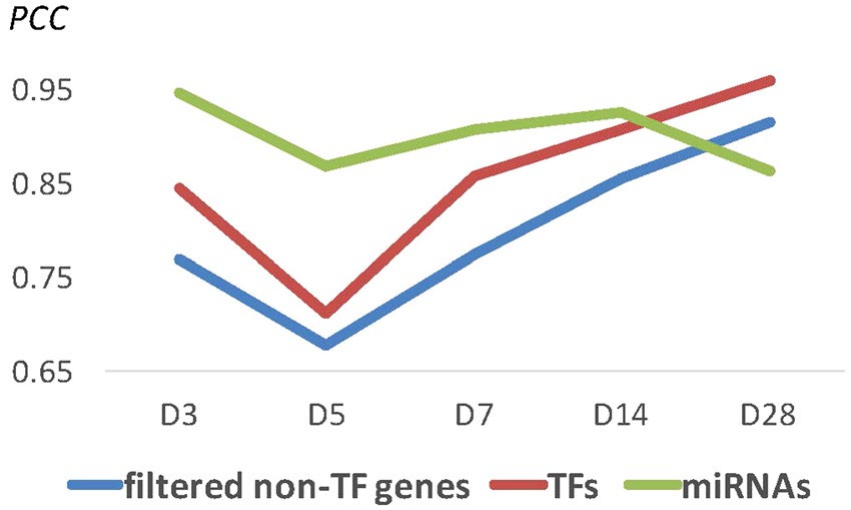
Pearson correlation coefficients (*PCCs*) of the expression intensity values of filtered non-TF genes (with expressional differences greater than 1 standard deviation but excluding those at the top and bottom 1% intensity values), TF genes, and microRNAs between with and without pressure overload in the cardiac samples at days 3, 5, 7, 14, and 28. The blue, red, and green lines represent the *PCC*s for filtered non-TF genes, TF genes, and microRNAs, respectively.

We first calculated the Pearson correlation coefficient (*PCC*) of gene expressions between sham and TAB at different time points. To reduce the bias that might arise from extremely high or low gene expression levels, those genes with expression intensity values above the top 1% or below the bottom 1% of all expressed genes at any time point were removed. Furthermore, to avoid the *PCC* being dominated by genes independent of the TAB treatment, the genes with expression differences between the two conditions that were not greater than one standard deviation in all time points were removed as well. Then, the *PCC*s of the total intensity values of the filtered non-TF genes (6,579 genes) between two different conditions were calculated for each time point (Suppl. Fig. S2). The same procedure was applied to 928 miRNAs and 606 TF genes to calculate their *PCC*s. For non-TF genes, the *PCC* between sham and TAB decreased from day 3 (0.77) to the minimum (0.68) at day 5 (blue line in Fig. 1). After day 5, the *PCC* increased from 0.78 at day 7, to 0.86 at day 14, and to 0.92 at day 28. The *PCC*s for TF genes showed similar tendencies: they were 0.84, 0.71, 0.86, 0.91, and 0.96 for days 3, 5, 7, 14, and 28, respectively (red line in Fig. 1). For miRNAs, the *PCC*s also decreased from day 3 (0.95) to day 5 (0.87), then slowly increased from 0.91 at day 7 to 0.93 at day 14, but decreased to 0.86 at day 28, which was similar to that for day 5 (green line in Fig. 1). Anatomically, the morphology of the heart after pressure overload still appeared normally at day 3. The heart size started to increase from day 5 to day 7 and became significantly larger in the TAB group (Suppl. Fig. S3A). The ratio of heart weight over body weight (HW/BW, an index of cardiac hypertrophy) remained similar between the TAB and sham groups at day 3 after surgery in mice and started to increase from day 5 to day 7 and reached a stable level at day 14 in TAB mice^20, 21^ (Suppl. Fig. S3B).

In summary, we made three observations (Fig. 1): (1) the *PCC*s for filtered non-TF genes and TF genes in murine hearts after pressure overload decreased from day 3 and reached the lowest at day 5. Then, the *PCC*s slowly returned to the same level as in the case without pressure overload. Therefore, day 5 is the critical transitional stage of gene regulation during the process of TAB-induced cardiac hypertrophy in male murine hearts. This observation is consistent with the result that most genes within the process of compensated hypertrophy returned to basal expression levels one week after TAB^16^. (2) The *PCC*s for all filtered non-TF genes and for TF genes showed similar tendencies of change, indicating that most non-TF gene expression changes may be directly or indirectly regulated by TFs. (3) For miRNAs, its *PCC* after day 5 did not return to the normal level as those for filtered non-TF genes and TF genes, but had a significant change at day 28. It seems that after day 5 the miRNA expression may have different regulatory mechanisms than those for filtered non-TF genes and TF genes.

### Functional enrichments of genes and miRNAs differentially expressed at early stages

The genes and miRNAs whose expression levels differed significantly between sham and TAB at least at one time point were defined as differentially expressed (DE) genes and miRNAs, respectively. We identified the DE genes and miRNAs from the whole transcriptome data for each time point and used the DE genes to predict their potential functions by Gene Ontology (GO) term enrichment analysis (see Methods).

**Figure 2:**
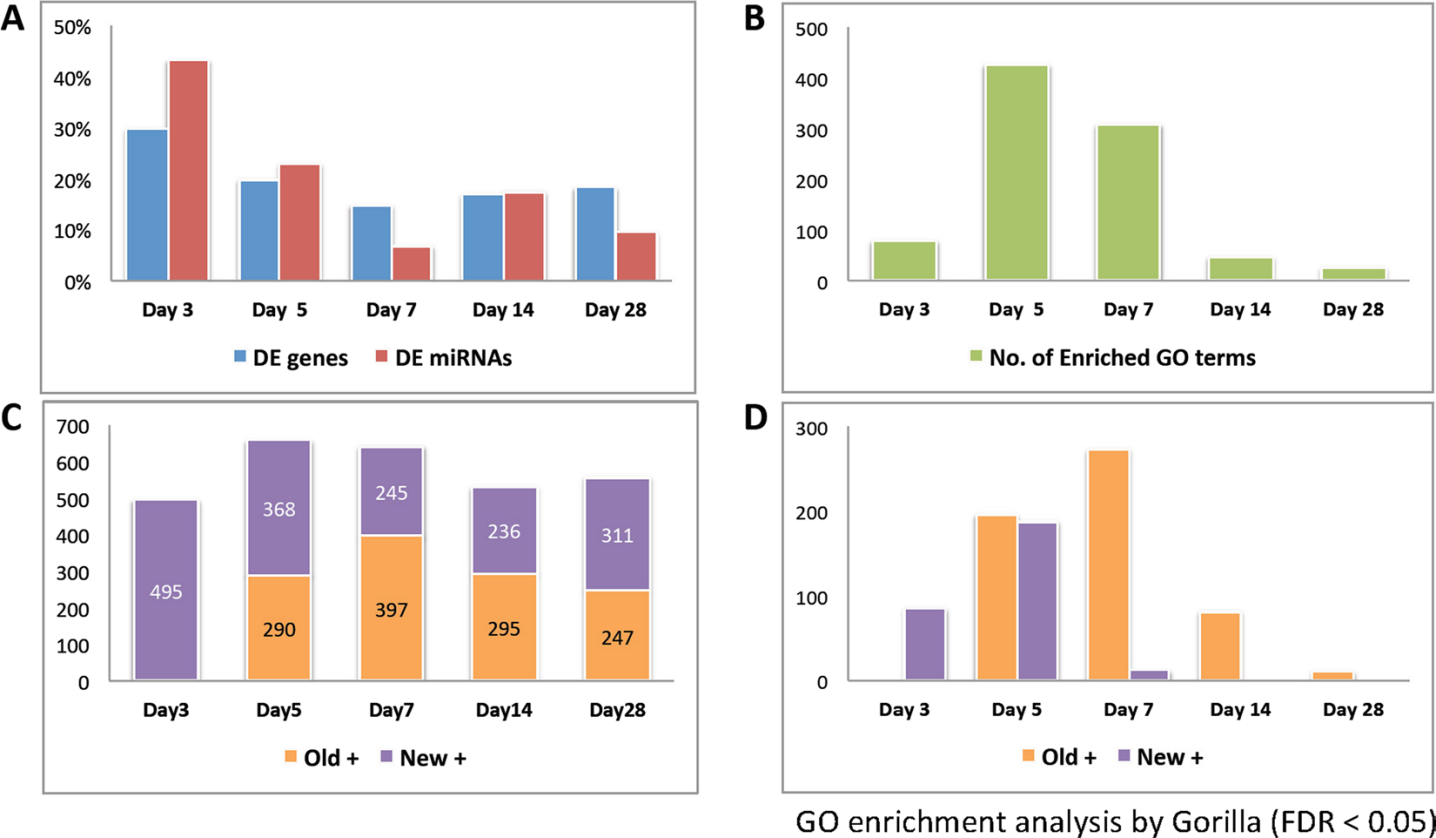
Proportions of differentially expressed (DE) genes and miRNAs and the number of enriched GO terms for DE genes at different time points. (**A**) Proportions of DE genes (blue bars) and miRNAs (red bars) that first time occurred at a time point. (**B**) Number of enriched GO terms for DE genes at a time point. (**C**) Numbers of up-regulated DE genes with first time up-regulation (New+ set; purple bars) at a time point and DE genes up-regulated at a previous time point (Old+ set; orange bars). (**D**) Numbers of enriched GO terms for DE genes from the New+ set (purple bars) and DE genes from the Old+ set (orange bars) at a time point.

We identified 2,853 DE genes and 148 DE miRNAs. There were 851, 917, 911, 908, and 948 genes and 64, 61, 67, 48, and 63 miRNAs differentially expressed at days 3, 5, 7, 14, and 28, respectively (Suppl. Fig. S4). There was no significant difference in the numbers of either DE genes or DE miRNAs between different time points. However, if we only considered the DE genes and miRNAs that were either up- or down-regulated for the first time at a specific time point, the proportions were 30%, 20%, 15%, 17%, and 18% of DE genes and 43%, 23%, 7%, 18%, and 9% of DE miRNAs at days 3, 5, 7, 14, and 28, respectively (Fig. 2A). Thus, half of DE genes and more than half of DE miRNAs arose for the first time at day 3 or 5. We confirmed some of the DE genes using quantitative real-time RT-PCR and the results showed that *Egr1*, *Egr2* and *cMyc* are indeed differentially expressed after TAB surgery at different time points (Suppl. Fig. S5).

Next, we inferred the enriched functions for identified DE genes. Using all identified DE genes as the queries and all expressed genes as the background (see Methods), we found 76, 30, and 15 enriched GO terms belonging to 20, 10, and 9 similar groups in the categories of biological process (BP), molecular function (MF), and cellular component (CC), respectively (Suppl. Table S1). The enriched functions for the up-regulated DE genes, including stimulus receptor on membrane, intracellular signal transduction, inflammatory response, and so on. These observations are mostly consistent with previous studies^22, 23^.

However, the numbers of enriched GO terms at different time points were not evenly distributed. We used the DE genes identified at each time point and inferred their enriched GO functions. After collecting the GO categories, we found 78, 426, 308, 46, and 25 GO terms enriched at days 3, 5, 7, 14, and 28, respectively (Fig. 2B). The numbers of enriched GO terms at days 5 and 7 were several times higher than those for the other three time points, although the numbers of DE genes between different time points did not differ significantly (Suppl. Fig. S4A and C). This result indicates that the DE genes at days 5 and 7 had more enriched functions related to pressure-overload induced cardiac hypertrophy.

We then asked whether the enriched functions were derived from the DE genes that were already up-regulated before or at that time point. We divided the up-regulated DE genes at a time point into two sets: (1) up-regulated for the first time at that time point; these genes were classified in the New+ set (purple bars in Fig. 2C) and (2) already up-regulated at a previous time point(s); these genes were classified in the Old+ set (orange bars in Fig. 2C). After the functional enrichment analysis, we found that the total numbers of enriched GO terms for the DE genes in the New+ and Old+ sets were almost the same in day 5, but those for day 7 were mostly from the Old+ set (Fig. 2D). That is, most of the enriched functions at day 7 were derived from the DE genes up-regulated at either day 3 or day 5 and those first up-regulated at day 7 did not seem to have strong functional preference. Thus, the DE genes that were up-regulated at an early time point (day 3 or 5) likely play a more specific role than those upregulated at a later stage in the process of TAB-induced cardiac hypertrophy in mice.

### Three TF coexpression modules regulate the genes with enriched functions related to cardiac hypertrophy

**Figure 3:**
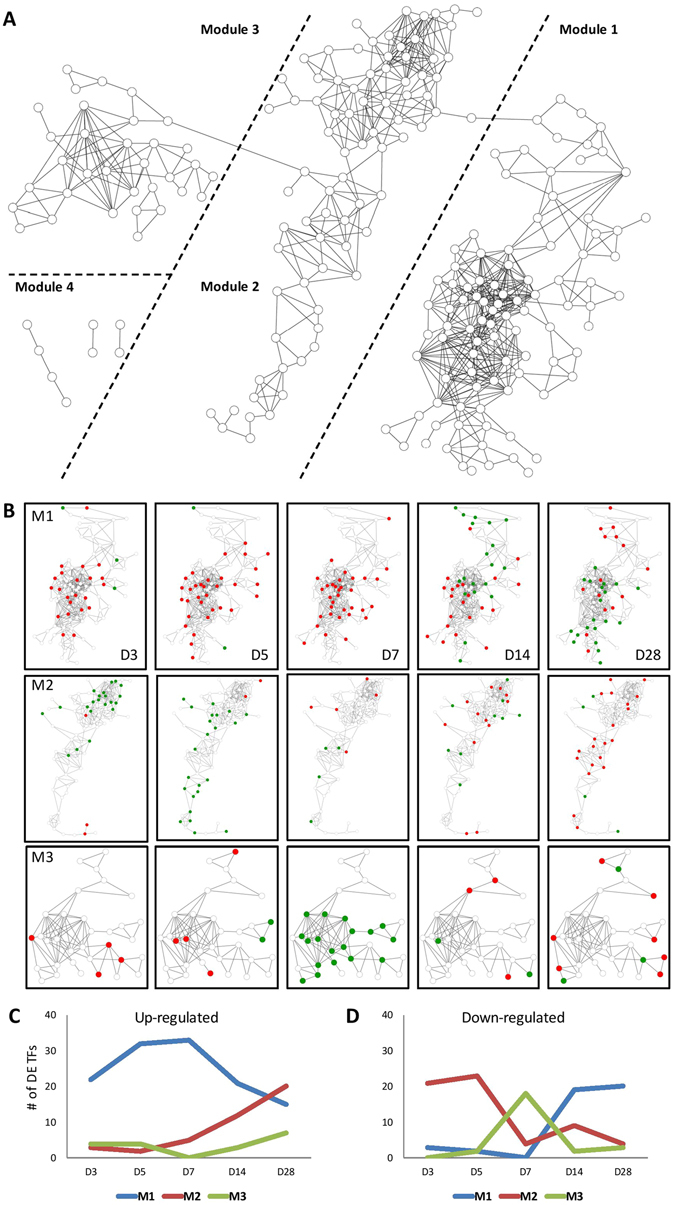
A positive gene coexpression network for differentially expressed (DE) TF genes under the TAB condition. (**A**) The four modules of TF genes in the network. (**B**) TF genes in Modules 1, 2 and 3 have different patterns of differential expression at each time point. The red and green nodes, respectively, indicate the up- and down-regulated TF genes at the time point. The line charts represent the numbers of (**C**) up-regulated and (**D**) down-regulated DE TF genes in Modules 1, 2 and 3 at different time points.

We used all DE TF genes and their expression profiles in TAB to construct a coexpression network in which each node represents a DE TF gene and an edge between two nodes represents a positive correlation (*PCC* ≥ 0.9) between the two DE TF genes. In the network, there were 206 DE TF genes each with at least one edge and the total number of edges was 670 (Fig. 3A). The network could be partitioned by a modified bridge-finding algorithm into three major modules, Modules 1, 2, and 3, which included 94, 79, and 33 TF genes, respectively (Fig. 3A; see Methods). In addition, there were 8 DE TF genes that had no connection with any TF gene in the first three modules; they were assigned to Module 4 and were ignored in the subsequent analysis.

We integrated the up and down regulation information at each time point into the modules (Fig. 3B), and found that Modules 1, 2 and 3 showed distinct gene up- and down-regulation patterns (numbers shown in Fig. 3C and D and proportions shown in Suppl. Fig. S6). In Module 1, most of the TF genes were up-regulated from days 3 to 7 and half of them were down-regulated after day 7 (Suppl. Fig. S6A). In Module 2, most of the TF genes were down-regulated at days 3 and 5 (Fig. 3D), but they began to be up-regulated after day 5 (Suppl. Fig. S6B). In Module 3, over half of the TF genes were down-regulated at day 7 (Suppl. Fig. S6C), while a few of the TF genes were up-regulated at other time points (Fig. 3C). That is, the number of up-regulated TF genes in Module 1 increased from day 3 to day 7 and then decreased, while those in Module 2 increased from day 14 to day 28 (Fig. 3C). For down-regulated TF genes, in contrast, the number in Module 1 increased from day 7 to day 14, while that in Module 2 decreased from day 3 to day 7 and that in Module 3 increased at day 7 and then decreased (Fig. 3D).

Next, the enriched functions for the TF genes and their coexpressed DE genes in each module were predicted. We first defined a DE gene as coexpressed with a TF gene if its intensity profile was positively correlated with that of the TF gene (*PCC* ≥ 0.9). After identifying the coexpressed genes, we conducted a functional enrichment analysis with GO-Slims, a cut-down version of the GO ontologies, for the three modules at each time point. We found 8, 28, 14, 11, and 14 GO-Slim terms enriched for days 3, 5, 7, 14, and 28, respectively, so the maximum number of enriched functions occurred at day 5 (Suppl. Fig. S7). The coexpressed DE genes in Module 1 had more enriched GO-Slim terms than those in the other two modules. Moreover, most of the GO-Slim terms for day 5 covered more than 75% of the total enriched functions for all time points. This result indicates that the DE genes coexpressed with the TF genes in Module 1, particularly those at day 5, may play a broader role than those in other modules during the hypertrophic process.

**Figure 4:**
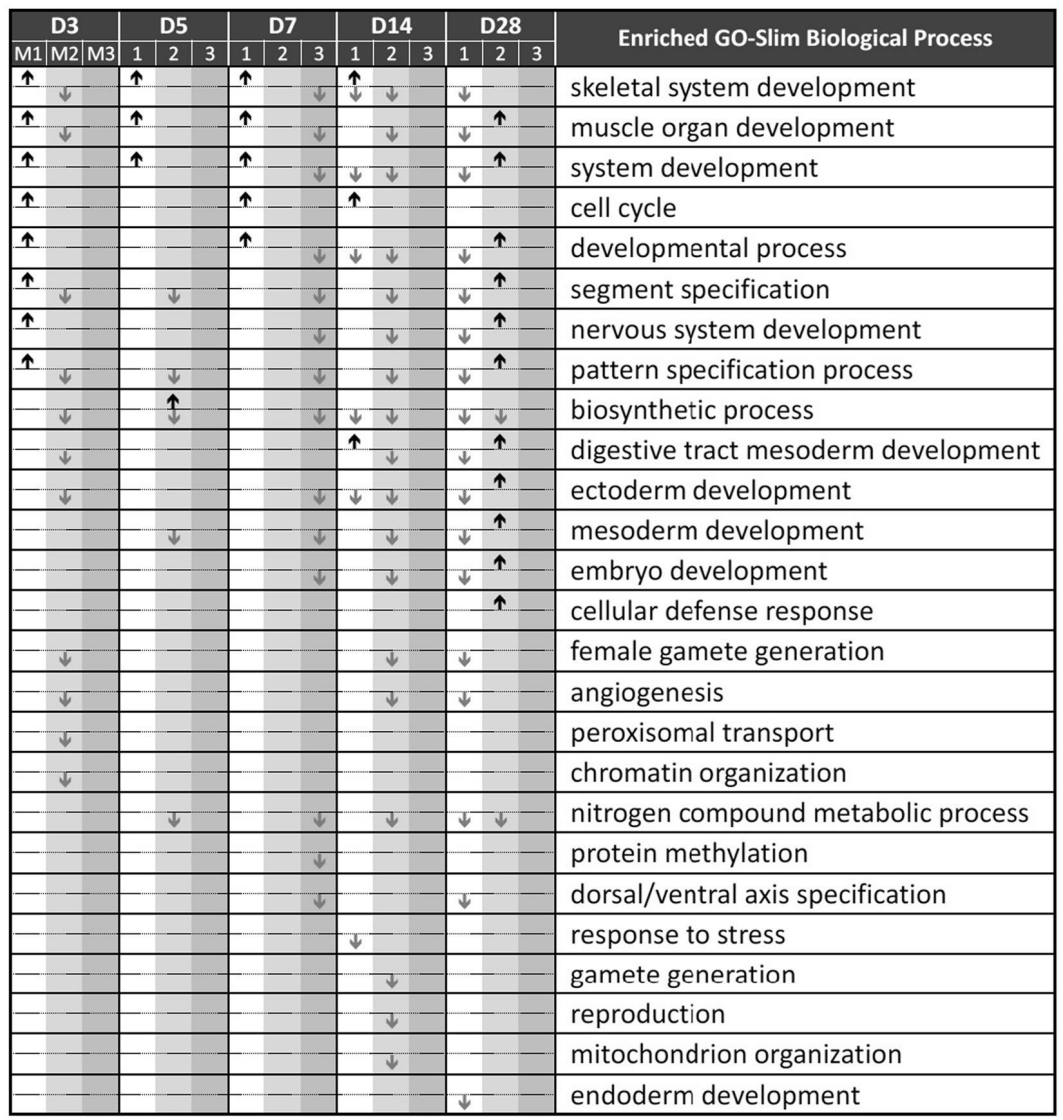
The enriched GO-Slim terms of TFs in the three modules at each time point. The major columns on left represent the five time points from day 3 to day 28 and the three sub-columns within each major column represent Modules 1, 2, and 3. The rows give the GO-Slim descriptions. The up and down arrows represent the GO-Slim in the row enriched (FDR < 0.05) for up- and down-regulated DE TF genes of the modules at the time point.

We also checked the enriched functions of TFs themselves for each module at each time point. To identify enriched functions of a TF in the network, we applied the GO-Slim enrichment analysis using all expressed genes as the background but excluding those genes related to fundamental transcriptional molecular-level functions (see Methods). For the TF genes in Module 1, the enriched functions, particularly for those up-regulated at day 3, included skeletal system, nervous system, and muscle organ development, segment specification, pattern specification process, and cell cycle (Fig. 4). However, only skeletal system, muscle organ, system development, and cell cycle can be found at the later time points in the same module and the enriched functions of digestive tract mesoderm development were found at day 14 only. In contrast, we could not find any enriched functions for the down-regulated TF genes in Module 1 from day 3 to day 7. In addition, almost all enriched functions found for up-regulated TF genes in Module 1 from day 3 to day 14 were also enriched for down-regulated TF genes at day 28 (Fig. 4), indicating that these enriched functions were up-regulated mostly at day 3 or at days 5, 7, and 14 but were all down-regulated at day 28. The transition in enriched functions from day 14 to day 28 could be explained by switching from up- to down-regulated TF genes in Module 1 (Fig. 3).

For the up-regulated TF genes in Module 2, only the function of biosynthesis process was enriched at day 5 but many important functions were enriched at day 28, including ectoderm, nervous system, muscle organ, embryo, and mesoderm development (Fig. 4). For the down-regulated TF genes in Module 2, most enriched functions were found at days 3 and 14 (Fig. 4). In contrast to Module 1, most of the up-regulated TF genes in Module 2 did not have enriched functions until day 28. At day 28, many enriched functions for down-regulated TF genes in Module 1 were also found for up-regulated TFs in Module 2, indicating that TF genes in Module 2 were recruited to regulate these functions in the later stage of pressure overload.

For TF genes in Module 3, we found no enriched function for the up-regulated TF genes at any time point, while many functions for down-regulated TF genes were found at day 7 (Fig. 4).

The above results reveal that the coexpressed TF genes in Modules 1 and 2 successively regulate the important functions associated with the cardiac hypertrophy. The TF genes in Module 1 probably responded to the pressure overload stimulus at the early stage (day 3) and continuously regulated some developmental functions until day 14. Then, the TF genes in Module 2 took over most of the functions and had four extra enriched functions at day 28. Finally, the TF genes in Module 3 had a few enriched functions from days 7 to 28.

### MiRNAs may suppress the expression of TF genes at different stages

We defined that a DE miRNA putatively suppresses the expression of a TF gene at a time point if the following three conditions are satisfied: (1) at least one binding site of the DE miRNA is found in the 3′UTR of the TF gene, (2) the miRNA is up-regulated at the time point, and (3) the TF gene is not up-regulated at that time point. According to this definition, we found that 69 miRNAs might down-regulate the expression of 130 TF genes at any of the five time points (Suppl. File S2). The numbers of the suppressing miRNAs and corresponding TF targets are 28, 37, 31, 22, and 44, and 93, 79, 87, 68, and 92 at days 3, 5, 7, 14, and 28, respectively (Suppl. Fig. S8). Because a miRNA can suppress multiple TF genes and a TF gene can be targeted by multiple miRNAs, the relationships of suppression from miRNAs to TF genes can be many-to-many.

We found that the up-down trend for the numbers of suppressing DE miRNAs was opposite to that for the number of suppressed TF gene targets at Day3 and day 5, but the same after day 7 (Suppl. Fig. S8). Based on this observation, we combined the analysis from the TF gene coexpression network of TF genes and miRNAs and hypothesized that the functions related to cardiac hypertrophy were induced by the up-regulated TF genes in Module 1 at days 3 and 5 but suppressed by some miRNAs after day 7. *Runt related transcription factor 1* (*Runx1*), for example, in Module 1 is a key regulator of heart post-myocardial infarction^24^ and was up-regulated from day 3 to day 14 and likely suppressed by some known cardiac miRNAs, such as miR-126–5p, miR-195, miR-208b, and miR-21 at day 28 (Suppl. File S2). As another example, *Cyclin E1* (*Ccne1*), a regulator related to defective cardiovascular development^25^, also in Module 1, was up-regulated at days 3, 7, and 14 and likely suppressed by miR-26b and miR-107 at day 28. Moreover, *Toll-like receptor 2* (*Tlr2*), a regulator involved in the signaling pathway of cardiomyocyte dysfunction and inflammatory responses^26^, also in Module 1, was up-regulated at day 7 and then likely suppressed by miR-208b at days 14 and 28.

### Gene Regulatory Network for Pressure Overload Cardiac Hypertrophy

Integrating our data with the target pair information from several interactome databases (see Methods), we constructed the regulatory relationships of DE genes by DE TF genes and DE miRNAs for pressure overload cardiac hypertrophy. The three relationships considered in this network were: (1) the regulatory pair of TF gene and target gene, (2) the regulatory pair of miRNA and target gene, and (3) the coexpressed pair of TF gene and miRNA (see Methods). In the last relationship, we did not consider the regulatory pair of TF gene and miRNA because only a few of the regulatory relationships have been identified so far. We identified 207 TF genes, 148 miRNAs, and 1,080 non-TF genes with the regulatory or coexpressed relationships in TAB. For the target DE genes, we analyzed their enriched functions and found 220 more enriched GO terms in addition to those for all DE genes (Suppl. File S3). Moreover, we also found that the proportion of cardiovascular-associated (CV) genes among the target genes (25%) was significantly higher than those among total DE genes (20%) and among all expressed genes (20%) (*p* < 0.01). These results suggest that the identified target DE genes in TAB and the associated regulators (TFs and miRNAs) likely play an important role for the induction of cardiac hypertrophy by pressure overload.

**Figure 5:**
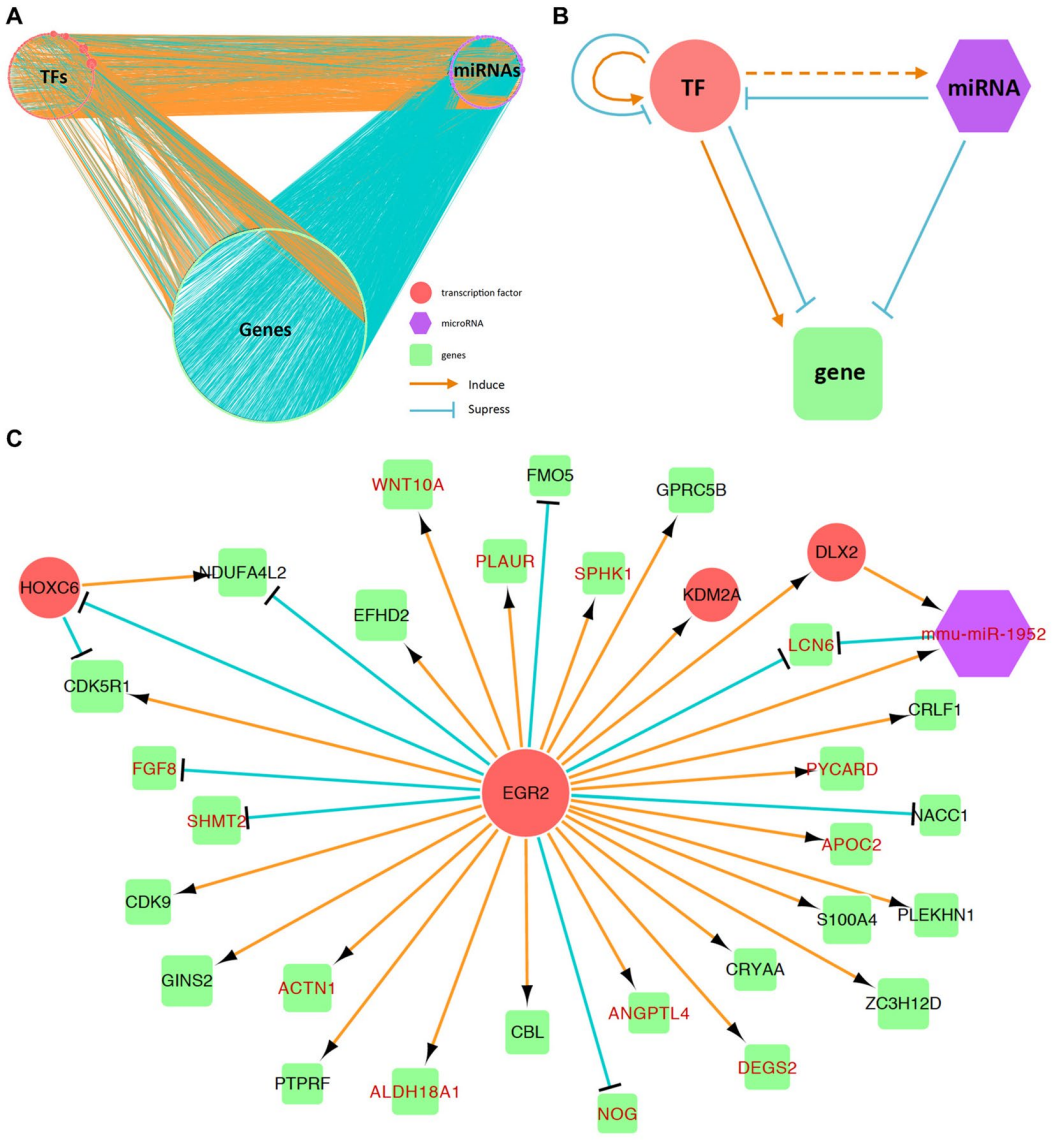
The gene regulatory network in TAB. (**A**) Network for all TF genes (red nodes), non-TF genes (green nodes), and miRNAs (purple nodes) and the edges of positive (orange) and negative (blue) regulations in TAB. (**B**) A schematic gene regulatory network for a TF gene, a miRNA, and a non-TF gene. (**C**) A sub-network centered at the TF *Egr2* gene to its direct targets, where genes with red name represent the cardiovascular-associated (CV) genes.

In addition, we integrated these relationships together and constructed a regulatory/coexpressed network. In the network, each node represents a gene, a TF gene, or a miRNA (Fig. 5A) and each edge represents one type of the three relationships between two nodes (Fig. 5B). For each pair of TF gene and target gene, the edge represents the positive or negative regulation from the TF gene to the target gene. For each pair of miRNA and gene, the edge represents the negative regulation from the miRNA to the target gene. For each pair of TF gene and miRNA, the edges from miRNA to TF gene and from TF gene to miRNA represent the negative regulation and positive coexpression, respectively. *Egr2*, an example shown in Fig. 5C, was one of the key regulators in TAB and had 30 target genes, including two TF genes, and was coexpressed with one miRNA. In addition, ~75% of the target genes were positively coexpressed with *Egr2*, indicating that *Egr2* is a positive regulator for most target genes.

To identify important regulators at each time point, we calculated the numbers of inward and outward edges and their sum for each regulator node. Then, the regulator nodes that first emerged at that time point were selected and sorted by the numbers of connecting edges. Table 1A and B show the connection and merged information for top 5 selected TF genes and top 3 selected miRNAs, respectively, at each time point. For example, *Runx1*, *Interleukin 6* (*Il6*), *Early growth factor 2* (*Egr2*), *Sex determining region y-box 9* (*Sox9*), and *T-cell leukemia homeobox 1* (*Tlx1*) were the top 5 TF genes that first emerged at day 3 and their total numbers of edges are 64, 41, 34, 24, and 21, respectively (Table 1A). Three of them, *Runx1*, *Il6*, and *Sox9*, have been reported as CV genes in previous studies^24, 27, 28^. In addition, at the same time point three first emerged miRNAs with the three largest numbers of connecting edges were miR-26b, miR-23b, and miR-98 (Table 1B), which all have been reported as relevant miRNAs in cardiac hypertrophy^29–31^.

**Table 1:** List of hubs in TAB-preferential regulatory network for each time point.

CV gene	TF Name	Number of edges	Outward (+, −)	Inward (+, −)	D3	D5	D7	D14	D28
**1**	*Runx1*	64	53 (43, 10)	11 (5, 6)	**1**	**1**	**1**	**1**	
**1**	*Il6*	41	37 (29, 8)	4 (3, 1)	**1**	**1**	**1**		**1**
	*Egr2*	34	32 (24, 8)	2 (2, 0)	**1**	**1**	**1**		**1**
**1**	*Sox9*	24	24 (19, 5)	0 (0, 0)	**1**	**1**	**1**	**1**	**1**
	*Tlx1*	21	21 (7, 14)	0 (0, 0)	**−1**		**−1**		
	*Pou2f2*	82	80 (64, 16)	2 (1, 1)		**1**	**1**	**1**	
	*Batf*	37	34 (30, 4)	3 (1, 2)		**1**	**1**	**1**	
	*Mxi1*	19	18 (7, 11)	1 (0, 1)		**−1**			
	*Sox10*	15	15 (13, 2)	0 (0, 0)		**1**			
	*Tgif1*	20	14 (13, 1)	6 (2, 4)		**1**	**1**		
**1**	*Vdr*	55	52 (43, 9)	3 (2, 1)			**1**		
**1**	*Irf7*	36	34 (33, 1)	2 (2, 0)			**1**		
**1**	*Pou2af1*	17	17 (11, 6)	0 (0, 0)			**1**		
**1**	*Rest*	17	17 (8, 9)	0 (0, 0)			**−1**		
**1**	*Nfe2*	14	13 (9, 4)	1 (0, 1)			**1**		
	*E2f1*	153	152 (127, 25)	1 (0, 1)				**1**	
**1**	*Egr1*	128	124 (99, 25)	4 (3, 1)				**−1**	
**1**	*Fos*	92	92 (80, 12)	0 (0, 0)				**−1**	
	*Rfx4*	19	18 (15, 3)	1 (1, 0)				**−1**	**−1**
**1**	*Rel*	19	17 (17, 0)	2 (0, 2)				**−1**	**−1**
**1**	*Gata1*	35	34 (30, 4)	1 (0, 1)					**−1**
	*Gli1*	39	34 (16, 18)	5 (0, 5)					**−1**
	*Zfp354c*	13	12 (8, 4)	1 (0, 1)					**−1**
**1**	*Egr3*	9	9 (8, 1)	0 (0, 0)					**1**
	*Prdm1*	9	6 (5, 1)	3 (1, 2)					**1**
	**miRNA ID**	**Number of edges**	**Outward**	**Inward**	**D3**	**D5**	**D7**	**D14**	**D28**
	mmu-miR-26b	78	70	8	**1**	**1**			**1**
	mmu-miR-23b	60	54	6	**1**	**1**	**1**	**1**	**1**
	mmu-miR-98	54	44	10	**1**	**1**	**1**	**1**	**1**
	mmu-let-7b/g/e	46	37	9		**1**			**1**
	mmu-miR-146a/b	22	18	4		**1**			**1**
	mmu-miR-130a	23	14	9		**1**			**1**
	mmu-miR-3963	14	10	4			**1**		
	mmu-miR-125a-5p	14	6	8			**1**		**1**
	mmu-miR-1224	9	7	2			**−1**		
	mmu-miR-208b	47	39	8				**1**	**1**
	mmu-miR-126-5p	42	31	11				**1**	**1**
	mmu-miR-130b	27	21	6				**1**	
	mmu-miR-497	58	48	10					**1**
	mmu-miR-499	45	36	9					**1**
	mmu-miR-152	34	29	5					**1**

(A) Top 5 TF genes (upper table) and (B) Top 3 miRNAs (lower table) with highest total degrees that first emerged at each time point. The values 1 and −1 in the five right most columns represent up- and down-regulated differential expression, respectively, from day 3 to day 28.

## Discussion

In this study, we analyzed the murine heart transcriptome data obtained at days 3, 5, 7, 14, and 28 under two conditions: with and without pressure overload. The expression levels were calculated for all expressed genes and miRNAs under the two conditions at the five time points and differentially expressed genes and miRNAs were identified. In addition, the expression profiles of the DE TF genes in TAB were used to infer their coexpression modules and predict potential functions. According to the targeting and coexpression information, the potential targets of the miRNAs and TF gene targets were identified and, finally, a TF gene-miRNA-gene regulation and coexpression network was constructed. Based on these results, we made the following observations that may answer questions related to the gene regulation in cardiac hypertrophy.

First, the *PCC*s for filtered non-TF genes, TF genes, and miRNAs between TAB and sham decreased from day 3, reached the lowest at day 5, and then returned to the same level at days 7 and 14 (Fig. 1). The global expression change was likely caused by many genes and miRNAs simultaneously in response to the pressure overload stimuli in the early stage, consistent with the previous study of pressure overload induced cardiac hypertrophy in mice^16^. Second, for miRNAs a globally significant transition occurred at day 28 (Fig. 1), indicating that miRNAs may play a regulatory role in cardiac hypertrophy at day 28. Third, the number of changes between suppressing DE miRNAs and that of suppressed DE TF gene targets showed the same trend only after day 7. We speculated that many DE TF genes were regulated by DE miRNAs in the later stages (Suppl. Fig. S8). Fourth, many CV TF genes in Module 1 were up-regulated at the early stages but down-regulated by miRNAs later (Suppl. File S2). Fifth, many functions related to cardiac hypertrophy were enriched in up-regulated DE TF genes in the early stages but enriched in down-regulated DE TF genes in the later stages (Fig. 4). From the last three observations, we may speculate that these functions were probably suppressed by DE miRNAs. Therefore, we hypothesize that TFs regulate genes and miRNAs in responding to the pressure overload stimuli in the early stages, and then miRNAs may regulate TF genes in a feedback way for maintaining the tissue homeostasis in the hypertrophic process.

In term of enriched GO functions for the DE genes, we found that the GO groups were mostly consistent with the general diagram of events found in the process of cardiomyocyte hypertrophy^22^, including hypertrophic stimulus response, intracellular signaling, and change in gene expression. In addition, many enriched GO groups were also associated with the event of hypertrophic stimulus response^22^, such as detection of stimulus (GO:0051606), response to stimulus (GO:0050896), sensory perception of chemical stimulus (GO:0007606), positive regulation of response to external stimulus (GO:0032103) in BP category, receptor activity (GO:0004872), ion channel activity (GO:0005216) in MF category and membrane part (GO:0044425), extracellular matrix (GO:0031012) in CC category. For the event of intracellular signaling, the GO similar groups such as G-protein-coupled receptor signaling pathway (GO:0007186) in the BP category, cytokine activity (GO:0005125), and molecular transducer activity (GO:0060089) in the MF category were overrepresented. Moreover, other GO groups regarding the gene regulation were enriched in our analysis, such as negative regulation of peptidase activity (GO:0010466), positive regulation of behavior (GO:0048520), cell proliferation (GO:0008284), immune system process (GO:0002684), and multicellular organismal process (GO:0051240) in BP category (Suppl. Table 1).

In term of specific genes as hypertrophic markers^32, 33^, some immediate-early genes, such as *Fos* and *Egr1*, and fetal genes, such as *Atrial natriuretic factor* (*Nppa*), *Beta-myosin heavy chain* (*Myh7*), and *Skeletal alpha actin* (*Acta1*) were also differentially expressed. However, many of these genes were not up-regulated or down-regulated at all time points. For example, *Myh7* was up-regulated at all time points except day 14, whereas *Egr1*, a TF, was down-regulated only at day 14. Thus, our data suggest that cardiac samples should be taken at several different time points over the time course in order, to have a reliable view of the gene expression profile over the time course.

The dynamic change of individual gene revealed in this study suggests critical time point of each gene playing its role to affect the progression of cardiac hypertrophy (Suppl. File. S1). For example, *Fkbp1b* modulates the calcium release from sarcoplasmic reticulum in cardiomyocyte and disruption of *Fkbp1b* results in cardiac hypertrophy in male mice^34^. In our results, during the developing stage, *Fkbp1b* was downregulated only before the hypertrophic growth of myocardium (day 3) and this suggests that downregulation of *Fkbp1b* at day 3 may be important to trigger the hypertrophic growth of heart. In contrast, expression pattern of *Egr1* was upregulated and reached maximal level in the early stage (day 5) and downregulated at day 14. Our finding suggests that *Egr1* participates in developing stage of cardiac hypertrophy but may not be required for the compensatory stage of hypertrophy. This may be reflected by the reduced cardiac hypertrophy in the *Egr1* knockout mice^35^. In addition, we found expression of *Gli1* was upregulated only at the late stage of cardiac hypertrophy (day 28). *Gli1* is a marker of perivascular mesenchymal stem cells and contributes to injury-induced organ fibrosis^36^. Removal of *Gli1* + cells reduces the pressure overload-induced cardiac hypertrophy and cardiac fibrosis^36^. The late induction of *Gli1* may reflect organ damage accumulated after long-term chronic pressure stress and also a hint of initiation of decompensation. Overall, our time domain-based quantification results can compensate the functional information derived from genetic modified mice studies.

Based on the targeting and coexpression relationships, we constructed a TF-miRNA-gene regulation and coexpression network and identified the hubs as potential candidates of essential regulators for cardiac hypertrophy (Table 1). Among the 25 TF candidates, 13 (~52%) of them are CV genes, which is significantly higher than the CV gene proportion in all expressed TF genes (22%), indicating that these detected TF genes are likely related to cardiac hypertrophy (Table 1A). For example, *Egr2*, not annotated as a CV gene yet, was differentially expressed at the beginning of TAB-induced cardiac hypertrophy and ~42% of its targets are CV genes, two of which, *Fibroblast growth factor 8* (*Fgf8*) and *Noggin* (*Nog*), have been reported to negatively regulate cardiac muscle tissue development^37, 38^. As *Egr1* and *Egr3*, two paralogous genes of *Egr2*, are both CV genes, and *Egr2* is likely a CV gene and an essential regulator for TAB-induced cardiac hypertrophy.

## Materials and Methods

### Animal model of cardiac hypertrophy and experimental design

Eight-week-old adult male mice (C57BL/6 J) weighing 20–25 g were anaesthetized (vaporized 1% isoflurane) and subjected to pressure overload by transverse aortic banding (TAB) according to the method described previously^18, 19^. Animals were kept under specific pathogen-free conditions, and all procedures performed were approved by the Institutional Animal Care and Use Committee (IACUC) of Academia Sinica, Taiwan. All experimental procedures were carried out in accordance with approved guidelines of the IACUC. The RNAs of cardiac tissues were taken from the left ventricles of Sham- and TAB-operated mice at 3, 5, 7, 14, and 28 days after surgery. Then, the expression changes of mRNAs and miRNAs were estimated by cDNA microarrays and miRNA arrays, respectively.

### Whole Genome OneArray® and miRNA OneArray®

#### Total RNA Isolation

Total RNA was extracted using the TRIzol® Reagent (Invitrogen, Carlsbad, CA) according to the manufacturer’s instructions. The concentration and purity of the RNA was measured by NanoDrop 1000 spectrophotometer (Thermo Fisher Scientific). Purity was checked by the ratio of the OD260/OD280 and OD260/OD230. The quality of total RNA was accessed using Agilent 2100 Bioanalyzer (Aglient Technologies, Santa Clara, CA, USA).

#### Mouse OneArray®

The Mouse Whole Genome OneArray® v2 (Phalanx Biotech Group, Taiwan) contains 27,307 DNA oligonucleotide probes, and each probe is a 60-mer designed in the sense direction. Among the probes, 26,423 probes correspond to the annotated genes in RefSeq v51 and Ensembl v65 database and the other 884 probes are used for control. The detailed descriptions of the gene array list are available at http://www.phalanx.com.tw/products/MOA.php.

Fluorescent RNA targets were prepared from 1 µg total RNA samples using OneArray® Amino Allyl aRNA Amplification Kit (Phalanx Biotech Group, Taiwan) and Cy5 dye (GE Healthcare). They were then hybridized to the Mouse Whole Genome OneArray® with Phalanx hybridization buffer using Phalanx Hybridization System. After 16 hrs hybridization, non-specific binding targets were washed away. The slides were scanned using a DNA microarray Scanner (Model G2505C, Agilent Technologies). The Cy5 Fluorescent intensities of each spot were analyzed by GenePix 4.1 software (Molecular Devices).

#### Mouse & Rat miRNA OneArray®

Mouse & Rat miRNA OneArray® v3 (Phalanx Biotech Group, Taiwan) contains triplicate 1086 unique miRNA probes from Mouse (miRBase Release 17) and 676 unique miRNA probes from Rat (miRBase Release 17). In addition, it also contains 144 experimental control probes. The detailed descriptions of the gene array list are available at http://www.phalanx.com.tw/products/MRmiOA_Probe.php.

Fluorescent targets were prepared from 2.5 µg total RNA samples using miRNA ULSTM Labeling Kit (Kreatech Diagnostics. USA) and then were hybridized to the Mouse & Rat miRNA OneArray® with Phalanx hybridization buffer using OneArray® Hybridization Chamber. After 16 hrs hybridization, non-specific binding targets were washed away. The slides were scanned using a DNA Microarray Scanner (Model 4000B, Molecular Devices, Sunnyvale, CA, USA). The Cy5 fluorescent intensities of each spot were analyzed by GenePix 4.1 software (Molecular Devices).

The microarray data have been submitted to the National Center for Biotechnology Information Gene Expression Omnibus database under accession number GSE99460.

#### Quantitative Real-time RT-PCR

The first-strand cDNA was synthesized from 1 μg of total RNA using the SuperScript III Reverse-Transcriptase Kit (Invitrogen). Real-time PCR reactions were performed in triplicate in 96-well plates with use of an ABI 7500 Real-Time PCR System instrument (Applied Biosystems). cDNA was amplified by the SYBR green PCR method (Kapa Biosystems). The relative expressions of *Egr1*, *Egr2* and *cMyc* genes were quantified by the comparative cycle threshold (Ct) method (∆∆Ct), with GAPDH as an endogenous control and normalized to day 3 sham. Sequences for primers were for mouse egr1, sense, 5′-TACGAGCACCTGACCACAGAGT-3′ and antisense, 5′-GCTGGGATAACTTGTCTCCACC-3′; egr2, sense, 5′-GTTGTGCGAGGAGCAAATGA-3′ and antisense, 5′-GCGAAGCTACTCGGATACGG -3′; cmyc, sense, 5′-AGCAGCGACTCTGAAGAAGAG-3′ and antisense, 5′-GAGACGTGGCACCTCTTGAG-3′; and for GAPDH, sense, 5′-CTTCACCACCATGGAGAAGG-3′ and antisense, 5′-GGCATGGACTGTGGTCATGAG-3′.

### cDNA and miRNA microarray data processing and normalization

For each cDNA array, the intensities were firstly normalized by median scaling normalization method, then taken the average of repeated data from the same sample. The whole data for a probe were removed if the intensity values were negative or less than one in any of the five time points. The remaining probe number was 20,021. The quantile approach was applied for the between-sample normalization. For each time point and condition data set, the intensity values were first sorted from the highest to the lowest. Second, for each condition the medium values of all intensity values at the same order positions were calculated. Finally, the normalized intensity values of each data set were re-assigned by the medium values of each corresponding sorted order position. If a gene had multiple probe sets, only that with the totally maximum absolute fold changes among all time points was selected to represent the expression profile of this gene. The final gene number for the analysis was 16,152. The Scatter Plots between TAB and sham gene expression levels at different time points were shown in Suppl. Fig. S2.

For the miRNA array data, the miRNAs with flags <0 within repeated probes and all chips were filtered out first. Then, the intensity values from repeated probes within one chip were combined by median. Then the combined values were normalized by a quantile normalized method within technical replicate. The processed data were provided by Phalanx Biotech Group, Taiwan. The number of remaining miRNAs was 928. The procedure for between-sample normalization is the same as that for cDNA array data^39^.

### TF and cardiovascular-associated gene information

The mouse TFs, transcription co-factors, and chromatin remodeling factors were collected from AnimalTFDB (http://www.bioguo.org/AnimalTFDB/) including 1,457 TFs of 71 families, 107 chromatin remodeling factors, and 279 transcription co-factors. Ignoring those without gene symbols, the final list included 1,740 TFs, transcription co-factors, and chromatin remodeling factors in total. The miRNA information was downloaded from miRBase (http://www.mirbase.org/) and the version was release 17. The cardiovascular GO Annotation Initiative gene list (comprising 4,054 genes) was assembled by merging 3 existing cardiovascular-associated (CV) gene lists (http://www.ebi.ac.uk/GOA/CVI) and downloaded from (ftp://ftp.ebi.ac.uk/pub/databases/).

### Identification of differentially expressed (DE) genes and miRNAs

For each time point, the fold-change of a gene was calculated by the log2 ratio of the intensity values measured from TAB-operated hearts over that measured from sham hearts. Then, the average and standard deviation of the total fold-change ratios for each time point were calculated. A differentially expressed (DE) gene was defined that its fold change was positively higher or negatively lower than two times of the standard deviation in any of the five time points. The same procedure was also applied to the miRNA array data.

### Correlation, statistical test, and functional enrichment analysis

The correlations of two intensity expression profiles were measured by Pearson correlation coefficient. The functional analysis tool used for the DE genes and genes coexpressed with DE TF genes in the network were GOrilla (http://cbl-gorilla.cs.technion.ac.il/) and PANTHER (http://www.pantherdb.org) that can identify enriched Gene Ontology (GO) and GO-Slim terms in ranked lists of genes against the background set of all expressed genes in this study. The species *Mus musculus* was selected and the false discovery rate (FDR) threshold was set to 0.05. The functional analysis tool used for the TFs themselves in the network was PANTHER against the background set of all expressed genes with the FDR threshold of 0.05 by the Benjamini-Hochberg procedure.

### Partition for the constructed TF coexpression network

An iterated bisection clustering method was applied to divide a connected network into multiple partitions step-by-step. First, all the bridges in the network were identified by a bridge-finding algorithm^40^. A bridge is an edge that when it is removed from a connected network, the network is divided into two separate networks. Second, the balancing values for the bridges with the size (number of nodes in the network) of the larger partition were calculated. Third, the bridge with smallest balancing value was selected as the cutting edge to divide the network into two partitions. Then the largest one from all partitions was chosen and divided further by repeating the method until no partition with size greater than 10 can be obtained, which is too few to do the functional enrichment test.

### Target gene prediction for TF and miRNA

The TF-target interaction data were integrated from two different sets: one predicted by Ernst *et al*.^41^, which had 468,319 interactions, and the other by Gerstein *et al*.^42^, which had 538,949 interactions. The original data of these two sets were both predicted in human. Using the ortholog mapping, both human data sets had been transferred as the mouse data set already^41, 42^. The two transferred mouse datasets were combined together. The miRNA-target interaction data was integrated from one experimental, miRTarBase (http://mirtarbase.mbc.nctu.edu.tw/), and two predicted datasets, TargetScanMouse Release 6.2 (http://www.targetscan.org/mmu_61/) and miRanda August 2010 release (http://www.microrna.org/microrna/). In miRTarBase Release 4.5, the number of miRNA-target interaction pairs for human was 38,113, where the numbers of miRNAs and target genes were 587 and 12,194, respectively. In contrast, the interaction number for mouse was only 9,378 where the numbers of miRNAs and target genes were 217 and 4,443, respectively. Using a similar procedure, the human data were converted to the mouse data by the ortholog mapping approach. If the miRNA and targets were both conserved in mouse, the pairs were also applied to the mouse analysis. In total, 48,466 miRNA-target pairs were collected from this data set. In the miRanda data set, 246,504 mouse miRNA-gene interactions had been predicted and were directly downloaded from the microcosm website (http://www.ebi.ac.uk/enright-srv/microcosm/) with *p*-value < 0.01, including 568 miRNAs and 19,183 target genes. In the TargetScanMouse dataset, both the conserved and non-conserved target site predictions were downloaded and the conserved predictions had 774 miRNAs and 11,828 genes and the non-conserved ones had 779 miRNAs and 18,249 genes. Finally, these three datasets were combined together and only the pairs of DE miRNAs and DE target genes were selected.

### TF-miRNA-gene regulatory and coexpression network construction

The TF-miRNA-gene network was constructed by considering the following three relationships: (1) the regulatory pair of TF gene and target gene, (2) the regulatory pair of miRNA and target gene, and (3) the coexpressed pair of TF gene and miRNA. For the first relationship, if a DE gene not only was coexpressed, either positively or negatively, with a DE TF gene in TAB but also had one or more of the DE TF gene predicted binding sites in the promoter region, they were defined as a regulatory pair of TF gene and target gene. For the second relationship, a regulatory pair of miRNA and target gene was defined as a DE gene that was not only negatively coexpressed with the DE miRNA in TAB but also a predicted target of that miRNA. For the third relationship, the coexpressed relationship of TF gene and miRNA, rather than the regulatory relationship, was considered because only a few of the TF-miRNA interactions were then available. The coexpressed pair of TF gene and miRNA was defined if a DE miRNA was positively coexpressed with a TF gene only in TAB. All networks were visualized by Cytoscape (http://www.cytoscape.org/).

## Electronic supplementary material

Supplementary Information

’Supplementary Dataset File 1

’Supplementary Dataset File 2

’Supplementary Dataset File 3
