# Supplementary material for: Three TF Co-expression Modules Regulate Pressure-Overload Cardiac Hypertrophy in Male Mice
Source: Sci Rep. 2017 Aug 8;7:7560. doi: 10.1038/s41598-017-07981-4 (PMC5548763; doi:10.1038/s41598-017-07981-4)
Supplement: Supplementary file 1 — Supplementary Information [file 41598_2017_7981_MOESM1_ESM.pdf]

Supplementary information

Three TF Co-expression Modules Regulate Pressure-Overload Cardiac Hypertrophy in Male Mice

Yao-Ming Chang[1], Li Ling[2], Ya-Ting Chang[2], Yu-Wang Chang[2], Wen-Hsiung Li[1,3],

Arthur Chun-Chieh Shih[4,*], and Chien-Chang Chen[2,*]

[1]Biodiversity Research Center and [2]Institute of Biomedical Sciences, Academia Sinica, Taipei, Taiwan, [3]Department of Ecology and Evolution, University of Chicago, Chicago, IL 60637, and [4]Institute of Information Science, Academia Sinica, Taipei, Taiwan

*: corresponding authors

Arthur Chun-Chieh Shih (email: arthur@iis.sinica.edu.tw)

Chien-Chang Chen (email: ccchen@ibms.sinica.edu.tw)

Supplementary Table S1. Overrepresented similar groups of GO terms for differentially expressed (DE) genes generated by Gorilla and REVIGO. BP, MF, and CC represent the GO categories of biological process, molecular function, and cellular component, respectively.

GO term ID (BP)	Description	$\log_{10}$(*p*-value)
GO:0007606	sensory perception of chemical stimulus	-15.87
GO:0007186	G-protein coupled receptor signaling pathway	-13.45
GO:0006952	defense response	-9.71
GO:0022610	biological adhesion	-9.31
GO:0007155	cell adhesion	-8.93
GO:0002684	positive regulation of immune system process	-8.82
GO:0002376	immune system process	-8.16
GO:0003008	system process	-7.16
GO:0051606	detection of stimulus	-7
GO:0060326	cell chemotaxis	-6.5
GO:0010466	negative regulation of peptidase activity	-5.6
GO:0044707	single-multicellular organism process	-5.45
GO:0051240	positive regulation of multicellular organismal process	-5.32
GO:0032501	multicellular organismal process	-5.18
GO:0030001	metal ion transport	-5.15
GO:0070098	chemokine-mediated signaling pathway	-4.88
GO:0030198	extracellular matrix organization	-4.8
GO:0043062	extracellular structure organization	-4.72
GO:0048520	positive regulation of behavior	-4.58
GO:0050896	response to stimulus	-4.44
GO:0008284	positive regulation of cell proliferation	-4
GO:0031341	regulation of cell killing	-3.5

GO term ID (MF)	Description	$\log_{10}$(*p*-value)
GO:0004872	receptor activity	-12.44
GO:0004984	olfactory receptor activity	-11.56
GO:0005539	glycosaminoglycan binding	-11.38
GO:0008201	heparin binding	-9.63
GO:0060089	molecular transducer activity	-9.42
GO:0005216	ion channel activity	-8.12
GO:0005125	cytokine activity	-7.28
GO:0005201	extracellular matrix structural constituent	-6.69
GO:0030414	peptidase inhibitor activity	-6.56

GO:1901681	sulfur compound binding	-4.5
GO:0001968	fibronectin binding	-3.45

**GO term ID (CC)**	**Description**	**$\log_{10}$(*p*-value)**
GO:0005576	extracellular region	-31.6
GO:0005578	proteinaceous extracellular matrix	-16.29
GO:0031012	extracellular matrix	-16.21
GO:0005887	integral component of plasma membrane	-10.29
GO:0005581	collagen trimer	-8.02
GO:0044459	plasma membrane part	-7.83
GO:0016021	integral component of membrane	-7.75
GO:0044298	cell body membrane	-3.6
GO:0044425	membrane part	-4

Supplementary Figures

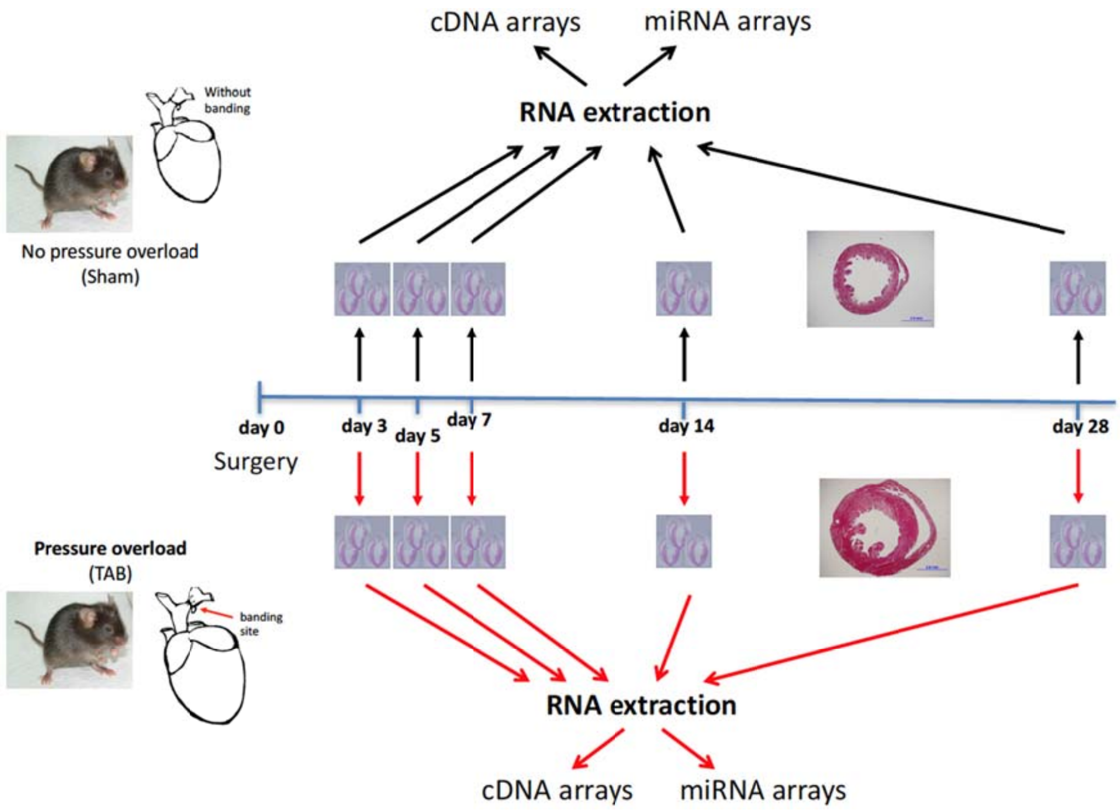

Figure S1. Design of the experiment. Eight-week-old adult male mice were subjected to pressure overload by transverse aortic banding (TAB). The RNAs of cardiac tissues were taken from mice subjected to TAB or sham operation at days 3, 5, 7, 14, and 28 after surgery. The expression level changes of mRNAs and miRNAs were measured by cDNA and miRNA microarrays, respectively.

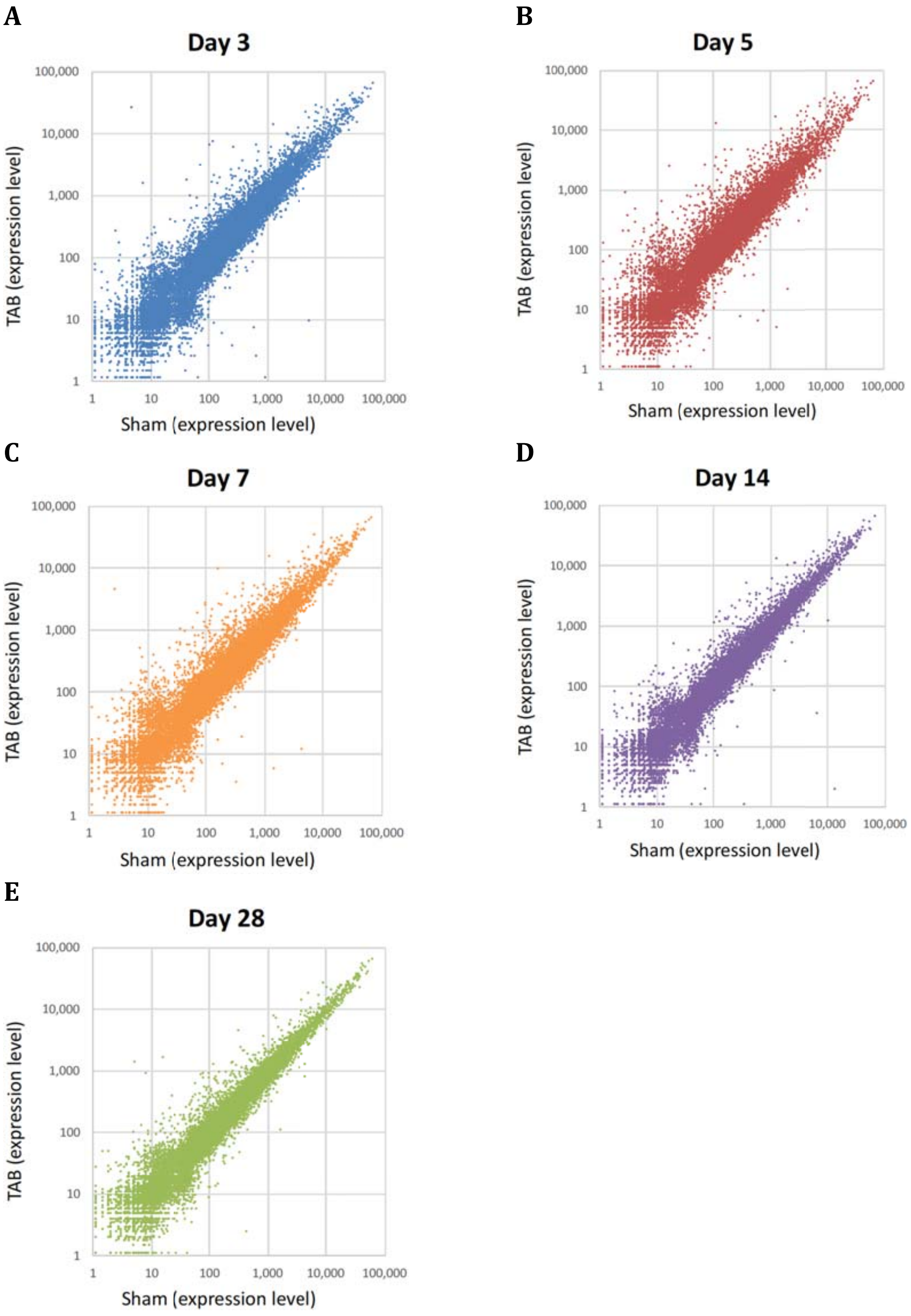

Figure S2. Scatter plots of TAB vs. sham gene expression levels at the five time points: (A) Day 3, (B) Day 5, (C) Day 7, (D) Day 14, and (E) Day 28. Each dot in the plot represents a gene with expression levels in sham (x-axis) and TAB (y-axis) conditions.

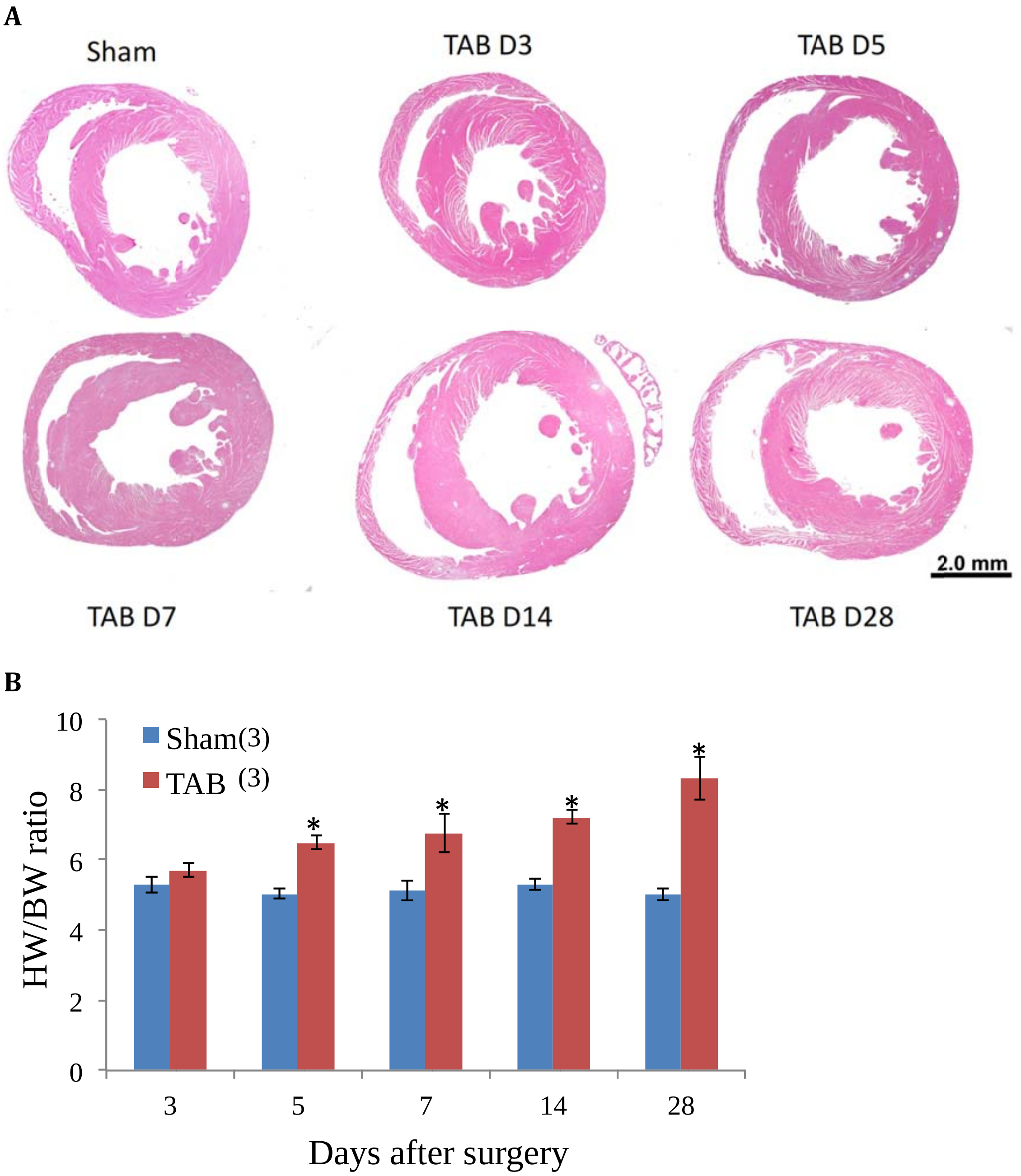

Figure S3. The cardiac hypertrophy in mice after TAB surgery. (A) Anatomic pictures of heart hypertrophy at different time points. (B) Heart weight/body weight (HW/BW) ratio of mice subjected to sham or TAB surgery after different time points. N=3 in each group, **p*<0.05 when compare to sham group.

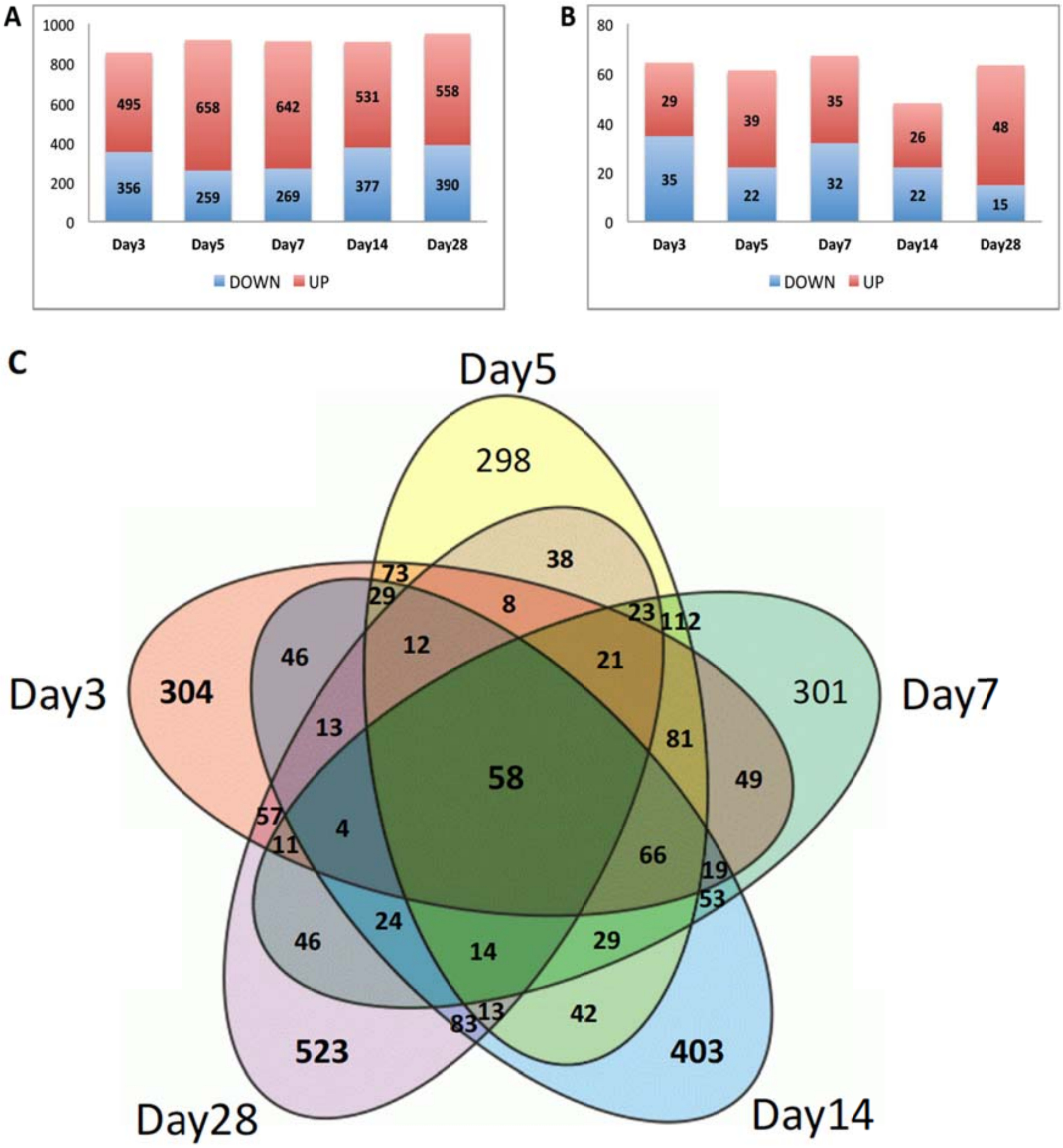

Figure S4. Numbers of differentially expressed (DE) genes and miRNAs at different time points. (A) Numbers of up- (red bars) and down-regulated (blue bars) DE genes at five time points. (B) Numbers of up- (red bars) and down-regulated (blue bars) DE miRNAs at five time points. (C) A Venn diagram to show the number of DE genes at five time points.

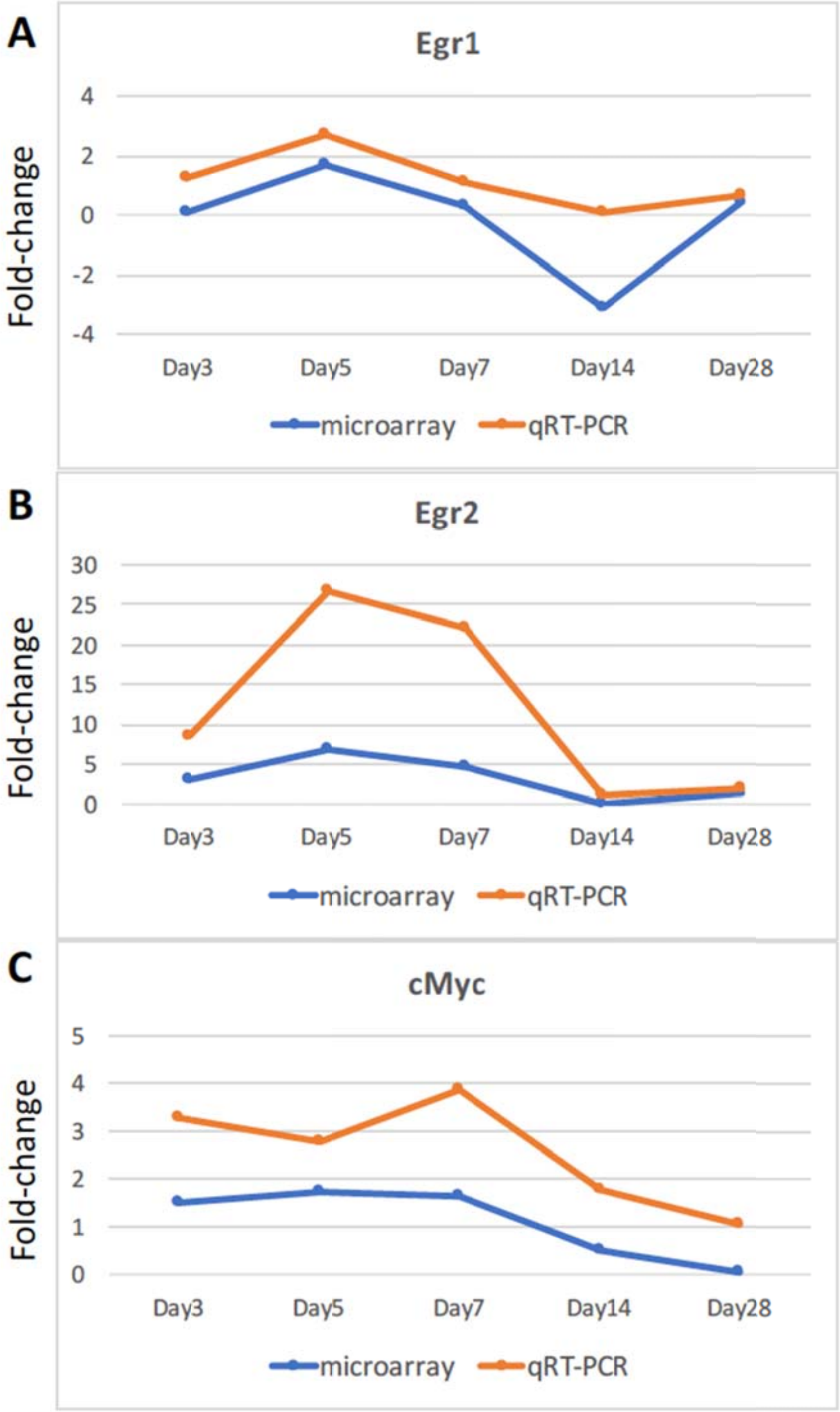

Figure S5. Fold-changes from expression levels comparing TAB and sham conditions for (A) *Egr1*, (B) *Egr2*, and (C) *cMyc*.

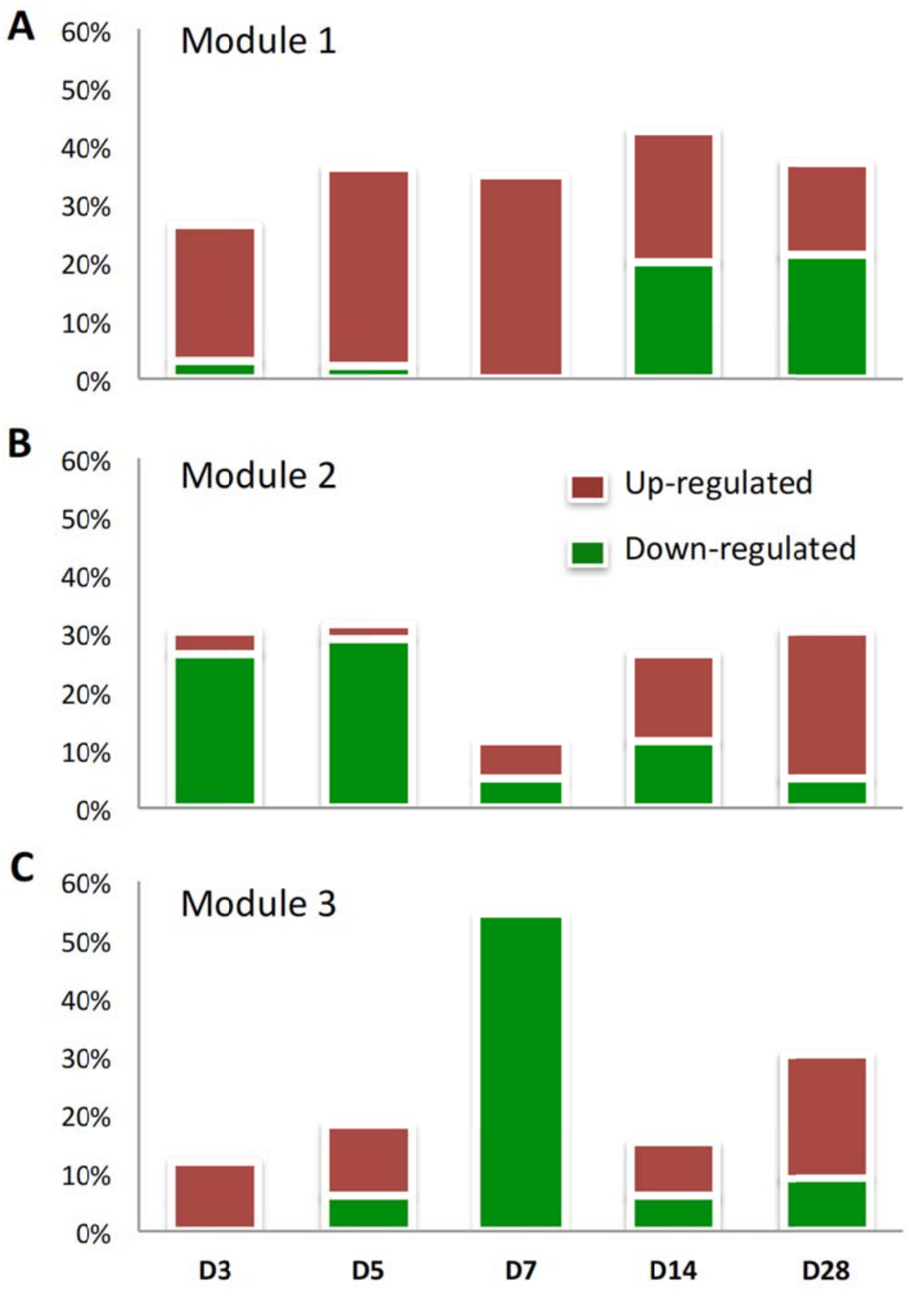

Figure S6. Proportions of up- and down-regulated DE TF genes in the three TF gene co-expression modules at five time points. (A) 94 TF genes in the Module 1, (B) 79 TF genes in the Module 2, and (C) 33 TF genes in the Module 3.

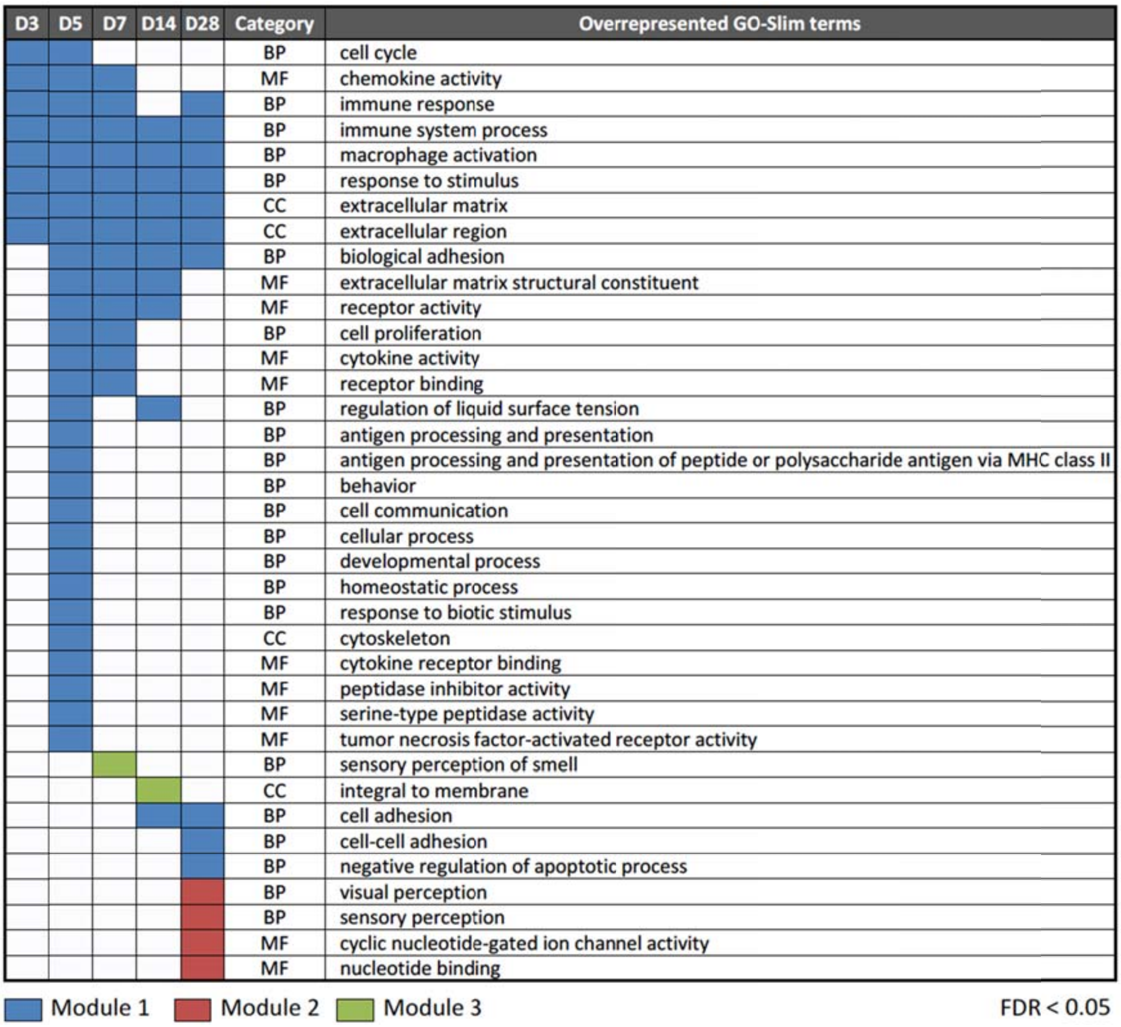

Figure S7. GO-Slim enrichment analysis for genes coexpressed with DE TF genes of the three modules at five time points. The columns on the left represent the time points. The rows represent the GO-Slim terms. We used blue, red, and green to indicate Modules 1, 2, and 3 in the GCN (Fig. 3A), respectively. The colored boxes represent the GO-Slim term of the row that is enriched at that time point of the column for genes coexpressed with DE TF genes in the module.

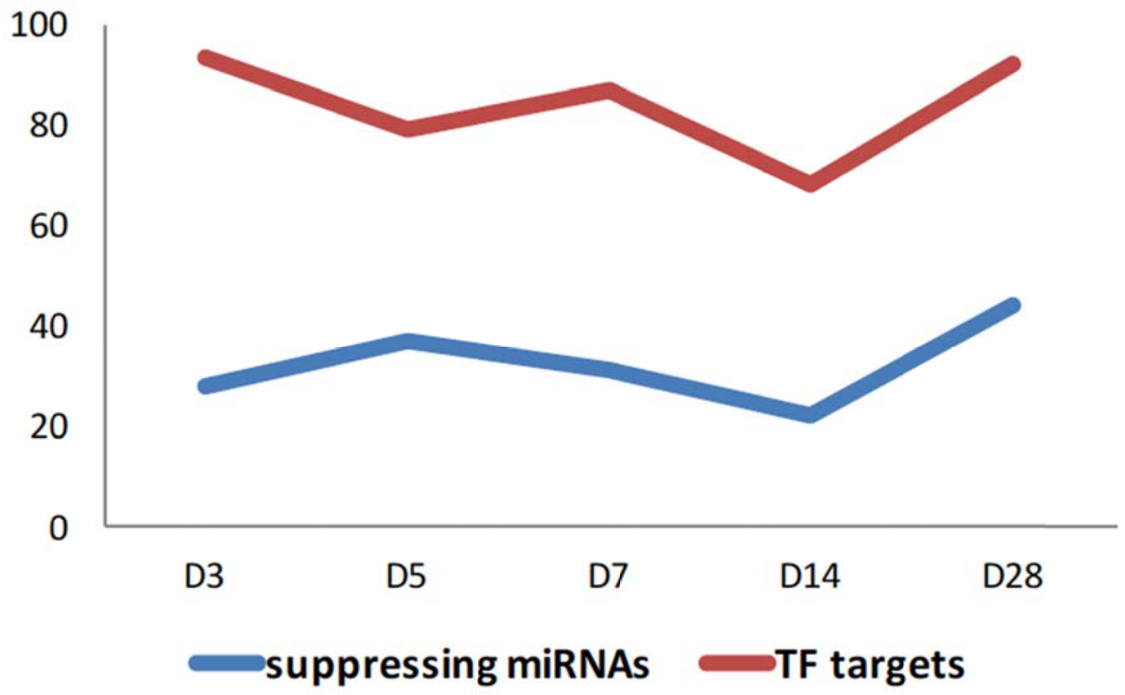

Figure S8. Numbers of suppressing miRNAs and suppressed TF gene targets of the three modules at five time points.
